# A guide to selecting high-performing antibodies for Protein-glutamine gamma-glutamyltransferase 2 (TGM2) for use in western blot, immunoprecipitation and immunofluorescence

**DOI:** 10.12688/f1000research.150684.2

**Published:** 2024-07-30

**Authors:** Riham Ayoubi, Maryam Fotouhi, Charles Alende, Sara González Bolívar, Kathleen Southern, Carl Laflamme, Neuro/SGC/EDDU collaborative group, ABIF consortium

**Affiliations:** 1 Department of Neurology and Neurosurgery, Structural Genomics Consortium, The Montreal Neurological Institute, McGill University, Montreal, Québec, H3A 2B4, Canada

**Keywords:** Uniprot ID P21980, TGM2, TGM, Protein-glutamine gamma-glutamyltransferase 2, antibody characterization, antibody validation, western blot, immunoprecipitation, immunofluorescence

## Abstract

Protein-glutamine gamma-glutamyltransferase 2 (TGM2) is a Ca ^2+^ dependent enzyme that catalyzes transglutaminase cross-linking modifications. TGM2 is involved in various diseases, either in a protective or contributory manner, making it a crucial protein to study and determine its therapeutic potential. Identifying high-performing TGM2 antibodies would facilitate these investigations. Here we have characterized seventeen TGM2 commercial antibodies for western blot and sixteen for immunoprecipitation, and immunofluorescence. The implemented standardized experimental protocol is based on comparing read-outs in knockout cell lines against their isogenic parental controls. This study is part of a larger, collaborative initiative seeking to address antibody reproducibility issues by characterizing commercially available antibodies for human proteins and publishing the results openly as a resource for the scientific community. While the use of antibodies and protocols vary between laboratories, we encourage readers to use this report as a guide to select the most appropriate antibodies for their specific needs.

## Introduction

Protein-glutamine gamma glutamyltransferase 2 (TGM2) belongs to the transglutaminase family of Ca ^2+^ dependent enzymes, regulating various cellular processes, including cell differentiation, growth and apoptosis. ^1^^–^^3^ Encoded by *TGM2* gene, TGM2 protein exhibits its function through Ca ^2+^-dependent cross-linking of substrates, thereby modulating their activity. ^1^ TGM2’s catalytic activity is dependent on guanine nucleotides and Ca ^2+^ binding. ^4^ Both GTP and/or GDP act as negative regulators of TGM2, inducing a conformational change upon binding, inhibiting its cross-linking activity (closed form). Conversely, Ca ^2+^ binding prompts a conformational change to induce TGM2 activity (open form). ^5^^–^^9^

Alteration and regulation of TGM2’s activity is associated with the pathogenesis of various diseases including cancer, neurodegeneration, fibrosis, inflammatory, autoimmune disorders and liver diseases. ^10^^–^^13^ Increased *TGM2* mRNA transcripts, resulting in elevated transaminase enzymatic activity, have been associated with neurodegenerative mechanism observed in Parkinson’s disease, Alzheimer’s disease and Huntington’s disease. ^14^^,^^15^ Given that α-synuclein serves as a common substrate for TGM2, elevated activity of TGM2 results in the formation of soluble aggregates as well as insoluble inclusions; distinctive features of Parkinson’s disease pathogenesis. ^15^^–^^18^ Regulating TGM2 activity using inhibitors could positively affect the human diseases in which TGM2 is implicated. ^19^^,^^20^ Identifying high-quality antibodies would accelerate TGM2 research and its potential as a pharmacological target.

This research is part of a broader collaborative initiative in which academics, funders and commercial antibody manufacturers are working together to address antibody reproducibility issues by characterizing commercial antibodies for human proteins using standardized protocols, and openly sharing the data. ^21^^–^^23^ Here, we evaluated the performance of seventeen commercially-available antibodies for TGM2 for use in western blot, and sixteen for immunoprecipitation and immunofluorescence, enabling biochemical and cellular assessment of TGM2 properties and function. The platform for antibody characterization used to carry out this study was approved by a committee of industry and academic researchers, whose members are mentioned in the competing interest section. It consists of first identifying appropriate human cell lines, development/contribution of equivalent knockout cell lines and finally following antibody characterization procedures on commonly used commercial antibodies. The standardized consensus antibody characterization protocols are openly available on Protocol Exchange, DOI: 10.21203/rs.3.pex-2607/v1. ^24^

## Results and discussion

Our standard protocol involves comparing readouts from wild-type (WT) and knockout (KO) cells. ^25^^,^^26^ The first step is to identify a cell line(s) that expresses sufficient levels of a given protein to generate a measurable signal. To this end, we examined the DepMap transcriptomics database to identify all cell lines that express the target at levels greater than 2.5 log _2_ (transcripts per million “TPM” + 1), which we have found to be a suitable cut-off (Cancer Dependency Map Portal, RRID:SCR_017655). Commercially available A549 cells expressed the TGM2 transcript at RNA levels above the average range of cancer cells analyzed. Parental and *TGM2* KO A549 cells were obtained from Abcam ( Table 1).

**Table 1.** Summary of the cell lines used.

Institution	Catalog number	RRID (Cellosaurus)	Cell line	Species of origin	Genotype
Abcam	ab275463	CVCL_0023	A549	Homo sapiens (Human)	WT
Abcam	ab261876	CVCL_B1I0	A549	Homo sapiens (Human)	*TGM2* KO

**Figure 1.**
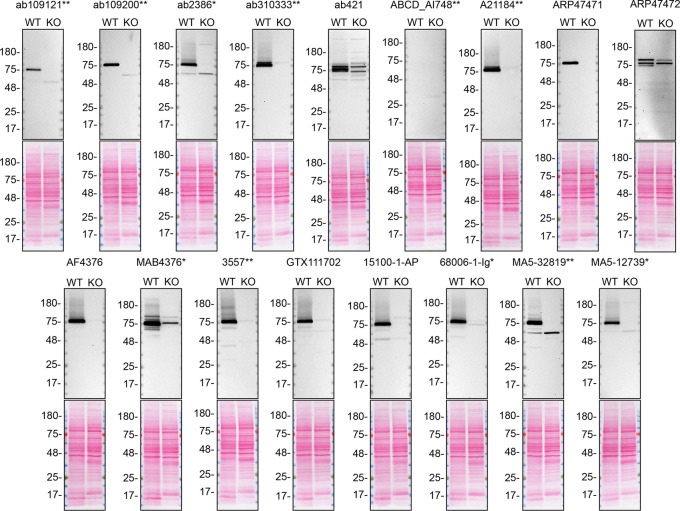
TGM2 antibody screening by western blot on lysate. Lysates of A549 (WT and *TGM2* KO) were prepared and 40 μg of protein were processed for western blot with the indicated TGM2 antibodies. The Ponceau stained transfers of each blot are presented to show equal loading of WT and KO lysates and protein transfer efficiency from the acrylamide gels to the nitrocellulose membrane. Antibody dilutions were chosen according to the recommendations of the antibody supplier. Antibody dilution used: ab109121** at 1/1000, ab109200** at 1/10000, ab2386* at 1/500, ab310333** at 1/1000, ab421 at 1/500, ABCD_AI748** at 1/10, A21184** at 1/10000, ARP47471 at 1/500, ARP47472 at 1/500, AF4376 at 1/400, MAB4376* at 1/200, 3557** at 1/500, GTX111702 at 1/500, 15100-1-AP at 1/6000, 68006-1-Ig* at 1/10000, MA5-32819** at 1/500, MA5-12739* at 1/200. Predicted band size: 77 kDa. *Monoclonal antibody, **Recombinant antibody.

**Figure 2.**
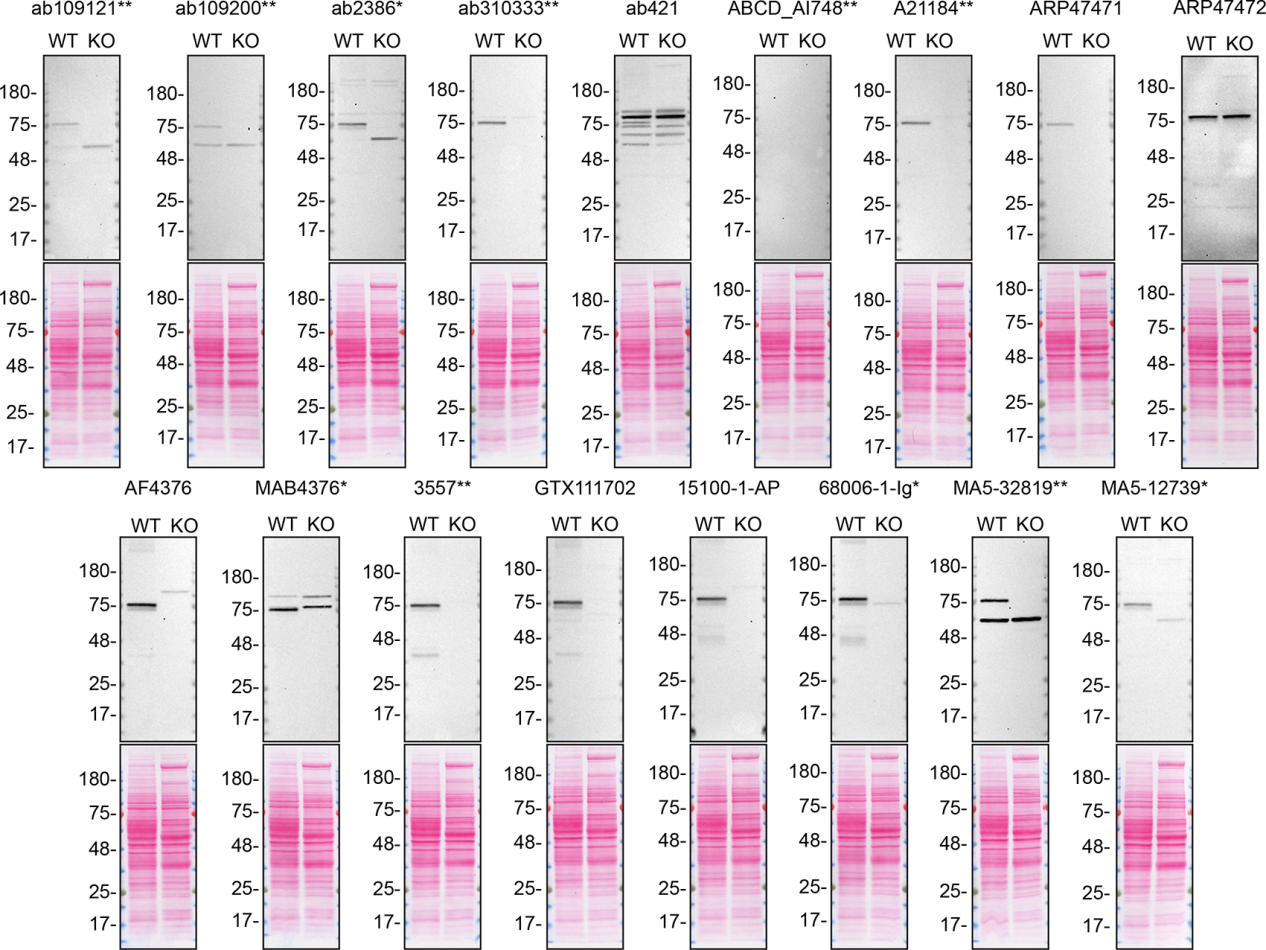
TGM2 antibody screening by western blot on culture medium. A549 WT and *TGM2* KO were cultured in serum free media, and 40 μg of protein from concentrated culture media were processed for western blot with the indicated TGM2 antibodies. The Ponceau stained transfers of each blot are shown. Antibody dilution used: ab109121** at 1/1000, ab109200** at 1/10000, ab2386* at 1/500, ab310333** at 1/1000, ab421 at 1/500, ABCD_AI748** at 1/10, A21184** at 1/10000, ARP47471 at 1/500, ARP47472 at 1/500, AF4376 at 1/400, MAB4376* at 1/200, 3557** at 1/500, GTX111702 at 1/500, 15100-1-AP at 1/6000, 68006-1-Ig* at 1/10000, MA5-32819** at 1/500, MA5-12739* at 1/200. Predicted band size: 77 kDa. *Monoclonal antibody, **Recombinant antibody.

For western blot experiments, WT and *TGM2* KO cells are collected as lysates or with a serum-free medium, ran on SDS-page, transferred on nitrocellulose membranes, and then probed with all antibodies in parallel ( Figures 1 and 2).

**Figure 3.**
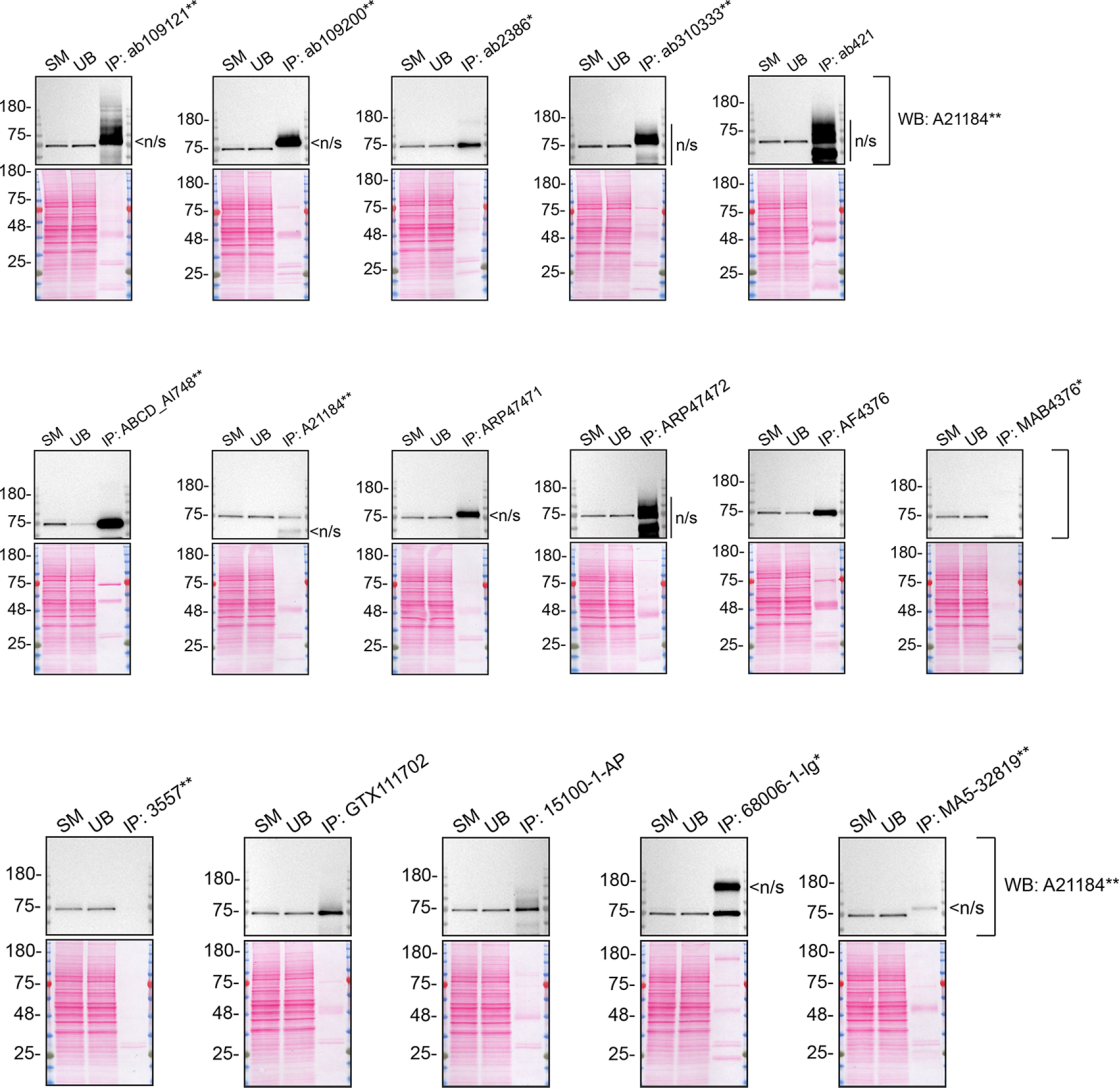
TGM2 antibody screening by immunoprecipitation on lysate. A549 lysates were prepared, and immunoprecipitation was performed using 2.0 μg of the indicated TGM2 antibodies pre-coupled to Dynabeads protein A or protein. Samples were washed and processed for western blot with the indicated TGM2 antibody. For western blot, A21184** was used at 1/10000. The Ponceau stained transfers of each blot are shown. SM=4% starting material; UB=4% unbound fraction; IP=immunoprecipitate; n/s=non-specific signal. *Monoclonal antibody, **Recombinant antibody.

As per our standard procedure, we next used the antibodies to immunoprecipitate TGM2 from A549 cell extracts. To evaluate the performance of each antibody, the TGM2 protein was detected in extracts, in each extract unbound to the antibody and corresponding immunoprecipitates (IP) ( Figure 3). To detect TGM2, a western blot was performed with an antibody successful under the conditions tested in Figure 1.

**Figure 4.**
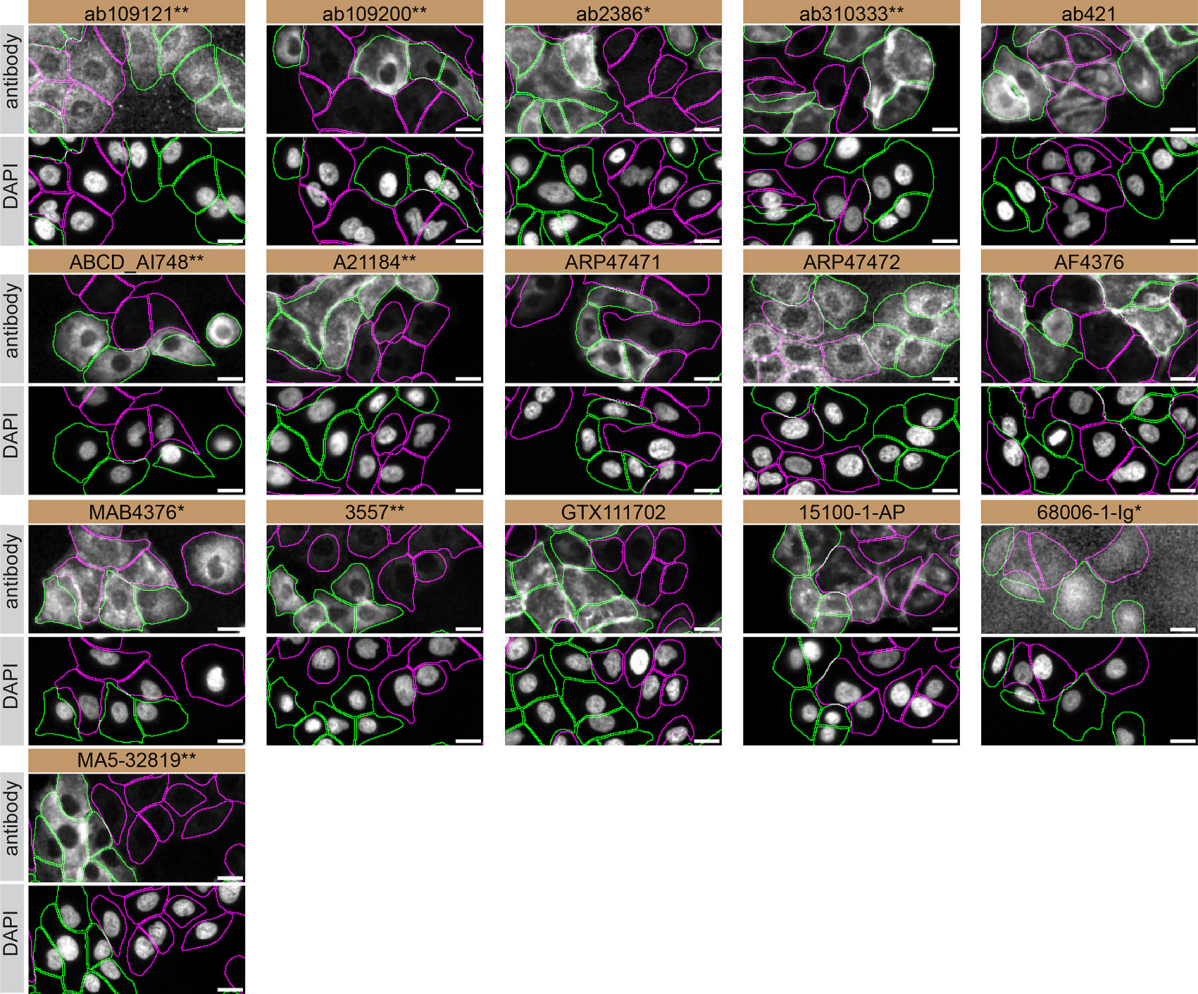
TGM2 antibody screening by immunofluorescence. A549 WT and *TGM2* KO cells were labelled with a green or a deep-red fluorescent dye, respectively. WT and KO cells were mixed and plated to a 1:1 ratio in a 96-well plate with optically clear flat-bottom. Cells were stained with the indicated TGM2 antibodies and with the corresponding Alexa-fluor 555 coupled secondary antibody including DAPI. Acquisition of the blue (nucleus-DAPI), green (identification of WT cells), red (antibody staining) and deep-red (identification of KO cells) channels was performed. Representative images of the merged blue and red (grayscale) channels are shown. WT and KO cells are outlined with green and magenta dashed line, respectively. When the concentration was not indicated by the supplier, which was the case for all antibodies tested, except ab310333**, ABCD_AI748 and A21184**, we tested antibodies at using the dilutions listed below. At these concentrations, the signal from each antibody was in the range of detection of the microscope used. Antibody dilution used: ab109121** at 1/1000, ab109200** at 1/300, ab2386* at 1/1000, ab310333** at 1/500, ab421 at 1/1000, ABCD_AI748** at 1/1000, A21184** at 1/1000, ARP47471 at 1/500, ARP47472 at 1/250, AF4376 at 1/100, MAB4376* at 1/100, 3557** at 1/500, GTX111702 at 1/1000, 15100-1-AP at 1/300, 68006-1-Ig* at 1/500, MA5-32819** at 1/1000. Bars = 10 μm. *Monoclonal antibody, **Recombinant antibody.

For immunofluorescence, antibodies were screened using a mosaic strategy, as per our standard procedure. First, A549 WT and *TGM2* KO were labelled with different fluorescent dyes in order to distinguish the two cell lines, and TGM2 antibodies were evaluated. Cells were imaged in the same field of view to reduce staining, imaging and image analysis bias ( Figure 4). Quantification of immunofluorescence intensity in hundreds of WT and KO cells was performed for each antibody tested. The images presented in Figure 4 are representative of the results of this analysis.

In conclusion, we have screened seventeen TGM2 commercial antibodies by western blot, and sixteen by immunoprecipitation, and immunofluorescence, comparing the signal produced by the antibodies in human A549 WT and *TGM2* KO cells. Several high-quality antibodies that successfully detect TGM2 under our standardized experimental protocol can be identified. Researchers who wish to study TGM2 in a different species are encouraged to select high-quality antibodies, based on the results of this study, and investigate the predicted species reactivity of the manufacturer before extending their research.

In our effort to address the antibody reliability and reproducibility challenges in scientific research, the authors recommend the antibodies that demonstrated to be underperforming under our standard procedure be removed from the commercial antibody market. Following the release of the antibody characterization, ab421 was removed from the manufacturer’s antibody catalog.

The authors do not engage in result analysis or offer explicit antibody recommendations. A limitation of this study is the use of universal protocols - any conclusions remain relevant within the confines of the experimental setup and cell line used in this study. Our primary aim is to deliver top-tier data to the scientific community, grounded in Open Science principles. This empowers experts to interpret the characterization data independently, enabling them to make informed choices regarding the most suitable antibodies for their specific experimental needs. Guidelines on how to interpret antibody characterization data found in this study are featured on the YCharOS gateway. ^27^

The underlying data for this study can be found on Zenodo, an open-access repository for which YCharOS has its own collection of antibody characterization reports. ^28^^,^^29^

## Methods

The standardized protocols used to carry out this KO cell line-based antibody characterization platform was established and approved by a collaborative group of academics, industry researchers and antibody manufacturers. The detailed materials and step-by-step protocols used to characterize antibodies in western blot, immunoprecipitation and immunofluorescence are openly available on Protocol Exchange, a repository dedicated to openly sharing scientific research protocols, DOI: 10.21203/rs.3.pex-2607/v1. ^24^

### Antibodies and cell line used

Cell lines used and primary antibodies tested in this study are listed in Table 1 and 2, respectively. To ensure that the cell lines and antibodies are cited properly and can be easily identified, we have included their corresponding Research Resource Identifiers, or RRID. ^30^^,^^31^

**Table 2.** Summary of the TGM2 antibodies tested.

Company	Catalog number	Lot number	RRID (Antibody Registry)	Clonality	Clone ID	Host	Immunogenic region	Concentration (μg/μl)	Vendors recommended applications	Number of citations ( CiteAb.com)
Abcam	ab109121**	1058833-3	AB_10861115	Recombinant-mono	EPR2956	rabbit	n/a	2.79	Wb	1
Abcam	ab109200**	1044092-1	AB_10860177	Recombinant-mono	EP2957	rabbit	proprietary information	0.30	Wb	12
Abcam	ab2386*	1051063-6	AB_2287299	Monoclonal	CUB 7402	mouse	proprietary information	n/a	Wb, IF	49
Abcam	ab310333**	1056093-5	AB_3076417	Recombinant-mono	EPR28142-86	rabbit	proprietary information	0.50	Wb, IF	0
Abcam	ab421	1034725-7	AB_304372	Polyclonal	-	rabbit	proprietary information	n/a	Wb, IF	37
ABCD	ABCD_AI748**	10/27/2023	AB_3076341	Recombinant-mono	679-14-E06	rabbit	proprietary information	0.12	Others	0
ABclonal	A21184**	3522042510	AB_3083448	Recombinant-mono	ARC52843	rabbit	aa 438-687	1.30	Wb, IF	n/a
Aviva Systems Biology	ARP47471	QC18320-43546	AB_1107120	Polyclonal	-	rabbit	middle region	0.50	Wb	n/a
Aviva Systems Biology	ARP47472	QC16720	AB_1088480	Polyclonal	-	rabbit	N-terminal	0.50	Wb	n/a
R&D Systems (a Bio-Techne brand)	AF4376	CFGU0119031	AB_10890213	Polyclonal	-	sheep	recombinant fragment (Ala2-Ala687)	0.20	Wb	4
R&D Systems (a Bio-Techne brand)	MAB4376*	CFNO0119031	AB_10971763	Monoclonal	716620	mouse	recombinant fragment (Ala2-Ala687)	0.20	Wb	2
Cell Signaling Technology	3557**	3	AB_2202883	Recombinant-mono	D11A6	rabbit	proprietary information	0.02	Wb	41
GeneTex	GTX111702	44524	AB_1952227	Polyclonal	-	rabbit	middle region	1.05	Wb, IF	8
Proteintech	15100-1-AP	00081307	AB_2202885	Polyclonal	-	rabbit	recombinant fragment (Ag7439)	0.60	Wb, IP, IF	21
Proteintech	68006-1-Ig*	10023724	AB_2918753	Monoclonal	2D4C11	mouse	recombinant fragment (Ag7462)	1.00	Wb, IF	n/a
Thermo Fisher Scientific	MA5-32819**	YJ4089240	AB_2810095	Recombinant-mono	JU30-02	rabbit	aa 578-627	1.00	Wb	0
Thermo Fisher Scientific	MA5-12739*	ZA4176225	AB_10985077	Monoclonal	CUB 7402	mouse	Purified guinea pig liver TGase II	0.20	Wb, IP, IF	115

Wb=western blot; IF=immunofluorescence; IP=immunoprecipitation, *=monoclonal antibody, **=recombinant antibody.

## Data Availability

Zenodo: Antibody Characterization Report for TGM2 (Protein-glutamine gamma-glutamyltransferase 2), https://doi.org/10.5281/zenodo.10819348. ^28^ Zenodo: Dataset for the TGM2 (Protein-glutamine gamma-glutamyltransferase 2) antibody screening study, https://doi.org/10.5281/zenodo.10927535. ^29^ Protocol Exchange: A consensus for antibody characterization platform, https://doi.org/10.21203/rs.3.pex-2607/v1. ^24^ Data are available under the terms of the Creative Commons Attribution 4.0 International license (CC-BY 4.0).
