# Supplementary material for: A guide to selecting high-performing antibodies for Protein-glutamine gamma-glutamyltransferase 2 (TGM2) for use in western blot, immunoprecipitation and immunofluorescence
Source: F1000Res. 2024 Jul 30;13:481. Originally published 2024 May 17. [Version 2] doi: 10.12688/f1000research.150684.2 (PMC11362715; doi:10.12688/f1000research.150684.2)
Supplement: Peer review history

# Reviewer report: Reviewer response for version 2

**DOI:** 10.5256/f1000research.169623.r316340

**Published:** 2024-08-29

**Authors:** Steve Jean

**Affiliations:** 1 Department of Immunology and Cell Biology, Université de Sherbrooke, Sherbrooke, Canada

In this data note, the authors evaluated a panel of commercial human transglutaminase 2 (TGM2) antibodies in three typical applications: immunoblotting, immunoprecipitation, and immunofluorescence. The manuscript is clear, and the data is well described. I am impressed that all the raw data can be easily accessed on the YCHAROS Zenodo website, which will ease evaluation by the community of the various antibodies tested. While this data note only refers to previously published protocols, the antibody report associated with this manuscript provides all the methodological information required to understand the data and to draw conclusions about the observed results. Previous comments from other reviewers were also well addressed or discussed adequately. Limitations of the standardized approaches are nicely discussed. I thus think that the manuscript is adequate for indexing.

 I only have a minor point that could further ease WB and IF data interpretation. It would be helpful to have the exposure time for the various WBs. This would provide another layer of information and ease the choice between two specific antibodies. Exposure time could be added at the bottom of each WB in Figures 1 and 2. Imaging parameters (or simply exposure time) could also be included in the legend of Figure 4. It is unclear if similar imaging parameters were used for all antibodies or if those were optimized for the best signal-to-noise ratio.

**Typo**: In the legend of Figure 3 (page 6), please add ‘G’ to the end of the sentence ‘… pre-coupled to Dynabeads protein A or protein.‘

Are sufficient details of methods and materials provided to allow replication by others?

Yes

Is the rationale for creating the dataset(s) clearly described?

Yes

Are the datasets clearly presented in a useable and accessible format?

Yes

Are the protocols appropriate and is the work technically sound?

Yes

Reviewer Expertise:

Membrane trafficking, RAB GTPases, phosphoinositides, Drosophila, proteomics, autophagy.

I confirm that I have read this submission and believe that I have an appropriate level of expertise to confirm that it is of an acceptable scientific standard.

# Reviewer report: Reviewer response for version 2

**DOI:** 10.5256/f1000research.169623.r308876

**Published:** 2024-08-21

**Authors:** Nicoletta Bianchi

**Affiliations:** 1 University of Ferrara, Ferrara, Italy

I have reviewed the changes made to the manuscript. I appreciate the inclusion of the number of times these antibodies have been reported in published articles, although the antibody panel has not been implemented. This still makes the widespread use of some of them by the scientific community. However, note that the citations have been reported in Table 2 and not in Table 1 as you indicated.

 Minor revisions have been modified as requested, as well as a clear description of the preparation of lysates was included.

 For this reason, I approve the manuscript in this form.

 Best regards

 Nicoletta Bianchi

Are sufficient details of methods and materials provided to allow replication by others?

Yes

Is the rationale for creating the dataset(s) clearly described?

Yes

Are the datasets clearly presented in a useable and accessible format?

Yes

Are the protocols appropriate and is the work technically sound?

Yes

Reviewer Expertise:

cancer research

I confirm that I have read this submission and believe that I have an appropriate level of expertise to confirm that it is of an acceptable scientific standard.

# Reviewer report: Reviewer response for version 1

**DOI:** 10.5256/f1000research.165276.r293962

**Published:** 2024-07-15

**Authors:** Nicoletta Bianchi

**Affiliations:** 1 University of Ferrara, Ferrara, Italy

Dear Editorial Team,

 I find this work extremely interesting. The authors tested the efficacy and specificity of commercial antibodies that can be used to study TGM2.

 Unfortunately, only one cell line and its KO were used, this is limiting, because the cell lines, especially cancer cells, can present different altered variants and/or different off-targets that could be recognized.

 At least the antibodies that are highly specific in this study could be tested on more cell lines. I believe that by contacting researchers who have developed TG2 KO lines or models, the authors could find a lot of willingness to collaborate.

 In any case, the manuscript is well done and of considerable applicative relevance and can help direct the researcher to a more appropriate choice of antibodies.

 However, for a better view of the applicability framework and before the indexing of this study, I suggest the inclusion of some antibodies from companies such as Zedira, which promotes a specific line of products for transglutaminase application, which are widely used by researchers working in the field of TGM2, as well as from Covalab.

 This would be of great interest and would greatly increase the impact of the work.

**Major revision:**

 The authors should implement the panel of antibodies including those widely used in publications on TGM2.

**Minor revision:**

**Keywords:** typos errors, punctuation errors, “western” in capital letters, as well as when reported in the manuscript

**Introduction**: “ *The protein* *s* *activity is dependent on cellular location, remaining inactive intracellularly, and becoming activated upon secretion*”, the sentence is incorrect, because it is also active inside the cells depending on calcium release stimuli.

 “ *by a committee of industry, academic research*”, which committee is this? must be specified.

 Some errors in the text.

**Results and discussion**: Authors should clarify the difference between the cell extract and lysate used. If they mean the same thing, it would be better to use the same term.

 With my best regards,

 Nicoletta Bianchi

Are sufficient details of methods and materials provided to allow replication by others?

Yes

Is the rationale for creating the dataset(s) clearly described?

Yes

Are the datasets clearly presented in a useable and accessible format?

Yes

Are the protocols appropriate and is the work technically sound?

Yes

Reviewer Expertise:

cancer research

I confirm that I have read this submission and believe that I have an appropriate level of expertise to confirm that it is of an acceptable scientific standard, however I have significant reservations, as outlined above.

# Response

**Published:** 2024-07-24

**Authors:** Kathleen Southern

**Affiliations:** McGill University, Canada

Dear Nicoletta Bianchi,

 Thank you for reviewing the article “A guide to selecting high-performing antibodies for Protein-glutamine gamma-glutamyltransferase 2 (TGM2) for use in western blot, immunoprecipitation and immunofluorescence” recently published to the YCharOS gateway. In response to your major and minor revisions ( *italicized*), the authors have edited the existing manuscript and will be submitting an updated version of the article shortly. We hope our response to your review clarifies any concerns or misunderstandings you may have previously had.

***Reviewer Comment -***

***Major revision:***

*The authors should implement the panel of antibodies including those widely used in publications on TGM2.*

**Author Response -**

 To address your major revision, in the newly submitted manuscript, we have included a column to Table 1 to indicated the number of times the tested antibodies were cited in published articles. The information is provided on CiteAb.com. Please visit the website if you’d like to replicate this study using other widely used antibodies that were not evaluated in this study.

***Reviewer Comment -***

***Minor revision***

***Introduction**: “The proteins activity is dependent on cellular location, remaining inactive intracellularly, and becoming activated upon secretion”, the sentence is incorrect, because it is also active inside the cells depending on calcium release stimuli.*

*“by a committee of industry, academic research”, which committee is this? must be specified.*

*Some errors in the text.*

**Author Response -**

 The sentence regarding the protein’s activity being associated to cellular location has been removed. As for the committee of industry and academic researchers, we have clearly indicated that the list of participating members is mentioned in the competing interest section

***Reviewer Comment -***

***Results and discussion**: Authors should clarify the difference between the cell extract and lysate used. If they mean the same thing, it would be better to use the same term.*

**Author Response -**

 In the second paragraph of this section, we have removed the word “extracts” to prevent misinterpretation and have clarified that cells were collected as lysates (medium free) or on culture medium to test the antibodies by western blot.

# Reviewer report: Reviewer response for version 1

**DOI:** 10.5256/f1000research.165276.r289998

**Published:** 2024-07-03

**Authors:** Michael A Rieger

**Affiliations:** 1 German Cancer Consortium (DKTK) and German Cancer Research Center (DKFZ), Goethe University, Cardio-Pulmonary-Institute, Frankfurt Cancer Institute, Frankfurt am Main, Germany; 2 Hospital of the Goethe University Frankfurt Department of Medicine II Hematology Oncology Rheumatology Infectious Diseases HIV Therapy, Frankfurt, Hesse, Germany

The authors of this study have tested 17 different commercially available antibodies against human Transglutaminase 2 (TGM2) on their specificity and feasibility for different biochemical and cell biological applications. They rigorously tested the antibodies for their suitability in Western blotting, immunoprecipitation and immune fluorescence. TGM2 is a multifunctional enzyme found in the cytosol and in the extracellular matrix. It functions as a glutaminase and a GDPase, depending on its conformation governed by the presence of Ca2+, thereby catalyzing peptide cross-linking and conducting signal transduction. TGM2 has been implicated in several diseases, such as celiac disease, cancer, neurodegenerative diseases, among others. Therefore, TGM2 has served as potential target for drug interference strategies in ongoing preclinical and clinical trials. The work presented here is of utmost interest for anybody working on the physiological and pathological function of TGM2, and provides solid data for selecting appropriate reagents to specifically and robustly detect TGM2 for research and diagnostic purposes. In general, work performed by the authors should be a prerequisite before using commercially available reagents (especially antibodies) to study biological effects and draw potentially wide-ranging conclusions which solely depend on the chosen diagnostic tool. Therefore, the manuscript here serves as a blueprint of how to test the validity of commercially available antibodies for several commonly used analytical methods.

 The authors provide here a most valuable resource of data, based on appropriately planned and conducted experiments. The manuscript is well written with high quality data representation, and the described methods are sound. The results shown here are of qualitative nature, and do not allow direct comparison of binding kinetics and sensitivity of the tested antibodies. Clear differences in the specificity, accuracy and suitability of the selected antibodies for the tested methods can be demonstrated, which will have consequences for evaluating and selecting TGM2 antibodies for future applications and studies, and for critical re-evaluation of findings from published studies. Last, vendors will decide on future continuation and/or recommendation of their products, based on these results.

 A few important minor points are still missing in this report, which the authors may want to address:

1. The sensitivity and specificity of antibodies depend on the correctly titrated amount and concentration used for the respective assay. The authors refer to their general protocol on how to test antibodies for their applications, but they do not provide an easy-to-see overview of the used concentrations of the presented TGM2 antibodies for each application. The authors may want to extend the information shown in Table 2 for each application presented in figures 1-4.
2. TGM2 is expressed in different isoforms, and at least four isoforms have been robustly reported and studied. These isoforms differ in their c-terminal part of the protein, and only isoform 2 is significantly shorter with reduced molecular weight that can be seen by Western. It would be of interest for the research community to learn about the isoform specificity of the tested antibodies. The authors should at least provide a table with the immunogens/peptides used to generate these antibodies. In the long run, the consortium should consider to test isoform specificity by overexpressing TGM2 isoforms in null cell lines.
3. Along the same lines, the authors should check for TGM2 mRNA expression of the different isoforms in their wt cell line A549 by isoform-specific qPCR. They should also briefly mention the different TGM2 isoforms and their functions in the introduction and point out the current limitations of their study regarding the isoform specificity. 

Are sufficient details of methods and materials provided to allow replication by others?

Partly

Is the rationale for creating the dataset(s) clearly described?

Yes

Are the datasets clearly presented in a useable and accessible format?

Yes

Are the protocols appropriate and is the work technically sound?

Yes

Reviewer Expertise:

Stem cells, Cancer stem cells, Single cell technologies, TGM2 physiology in colorectal cancer and other cancers

I confirm that I have read this submission and believe that I have an appropriate level of expertise to confirm that it is of an acceptable scientific standard, however I have significant reservations, as outlined above.

# Response

**Published:** 2024-07-17

**Authors:** Kathleen Southern

**Affiliations:** McGill University, Canada

Dear Michael A. Rieger,

 The authors would like extend our gratitude for having taken the time to review this article and provide concrete feedback. Please see our response to your points ( *italicized*) which we have responded to and/or addressed in a new version of the article, which will be submitted to F1000 shortly. We hope to have clarified any concerns or misunderstandings you previously had and believe the article is now ready for approval.

***Reviewer Comment:***

*1. The sensitivity and specificity of antibodies depend on the correctly titrated amount and concentration used for the respective assay. The authors refer to their general protocol on how to test antibodies for their applications, but they do not provide an easy-to-see overview of the used concentrations of the presented TGM2 antibodies for each application. The authors may want to extend the information shown in Table 2 for each application presented in figures 1-4.*

**Author Response:** We understand how the concentration of antibodies used in each application were not clearly accessible in the report. Based on our in depth procedure referred to in methods section ( https://protocolexchange.researchsquare.com/article/pex-2607/v1)  and the dilutions presented in the Figure legends, we can assume the viewers can determine which concentration was used for the respective assays or are able to reproduce the experiment based on the information provided.

***Reviewer Comment:***

*2. TGM2 is expressed in different isoforms, and at least four isoforms have been robustly reported and studied. These isoforms differ in their c-terminal part of the protein, and only isoform 2 is significantly shorter with reduced molecular weight that can be seen by Western. It would be of interest for the research community to learn about the isoform specificity of the tested antibodies. The authors should at least provide a table with the immunogens/peptides used to generate these antibodies. In the long run, the consortium should consider to test isoform specificity by overexpressing TGM2 isoforms in null cell lines.*

**Author Response:** We understand this need and have added a column to table 2 to provide the readers with the immunogenic or peptide region the antibodies target, based on the information provided on the antibody manufacturer catalog.

***Reviewer Comment:***

*3. Along the same lines, the authors should check for TGM2 mRNA expression of the different isoforms in their wt cell line A549 by isoform-specific qPCR. They should also briefly mention the different TGM2 isoforms and their functions in the introduction and point out the current limitations of their study regarding the isoform specificity.*

**Author Response:** The authors are aware of that TGM2 isoforms exists, but that being said the purpose of this Data note is not to provide information regarding those isoforms but rather deliver a toolkit, to be used by researchers who wish to study TGM2 in relation to health and/or disease, by demonstrating which commercially available antibodies are of high-quality to be selected and used to enhance their research. Using these antibodies, these researchers can dive deeper and decipher whether the isoforms are present in the cell line. This level of detail is outside the scope of this initiative, which follows a platform (see link to protocol exchange preprint above) to validate antibodies by creating/obtaining knockout (KO) cell lines for a protein target and screening all commercial antibodies against that target head-to-head in key academic applications, comparing wild-type to KO cells.
